# Effectiveness of a Co-Production with Dialogue Program for Reducing Stigma against Mental Illness: A Quasi-Experimental Study with a Pre- and Post-Test Design

**DOI:** 10.3390/ijerph192114333

**Published:** 2022-11-02

**Authors:** Eiichi Nakanishi, Masahiro Tamachi, Takeshi Hashimoto

**Affiliations:** 1 Department of Occupational Therapy, Faculty of Health Sciences, Bukkyo University, 7 Nishinokyo, Higashi-toganoo-cho, Nakagyo-ku, Kyoto 604-8418, Japan; 2 Department of Physical Therapy, Faculty of Health Sciences, Aino University, 4-5-4 Higashi-Ohda, Ibaraki City 567-0012, Japan; 3 Graduate School of Health Sciences, Kobe University, 7-10-2 Tomogaoka, Suma-ku, Kobe 654-0142, Japan

**Keywords:** stigma reduction, co-production with dialogue, health science students, people with mental illnesses

## Abstract

For people with mental illnesses, stigma represents a barrier to social participation. Health professionals, including students, often hold stigma toward such individuals, Further, people with a mental illness often have self-stigma. This study aimed to both develop and examine the effectiveness of a new program based on co-production with dialogue for reducing stigma among both health science students and people with mental illnesses. This was a quasi-experimental study, with a pre- and post-test design and no control group. The sample comprised 28 university students majoring in occupational therapy and 20 community-dwelling people with mental illnesses. The Co-Production with Dialogue Program for Reducing Stigma (CPD-RS) was administered to this sample. Link’s Devaluation Discrimination Scale (DDS) was used to assess whether the program reduced stigma. Compared to their preintervention scores, the students’ postintervention DDS scores significantly decreased, persisting for at least one month, but those of people with mental illnesses showed no significant change. Both the students and the people with mental illnesses evaluated the program as “positive” through a questionnaire administered two months after the intervention. These results suggest that the CPD-RS reduces health science students’ stigma toward people with mental illnesses and fosters mutual understanding between the two groups.

## 1. Introduction

The Greeks created the term “stigma” to describe “bodily signs designed to expose something unusual and bad about the moral status of the signifier” [1]. Ample research has examined the psychological aspects of stigma, including its definitions, conceptualizations, dimensions, and processes [2,3,4].When applied to mental illness, stigma refers to social judgment, degradation, or devaluation of individuals because they exhibit symptoms of mental illness or have been labeled as having a mental illness [3]. Stigma has the following three key components: (1) knowledge-related issues (ignorance), (2) attitudinal issues (prejudice), and (3) behavioral issues (discrimination) [4]. These issues can precipitate social exclusion; thus, stigma may make it difficult for people with a mental illness to have productive and fulfilling lives [5,6]. There are two types of mental illness stigma, namely public stigma and self-stigma. Public stigma refers to the general public’s discriminatory response to people with mental illnesses. Self-stigma is the internalization of public stigma regarding mental illness [3].

Medical practitioners hold varying attitudes toward people with mental illnesses [7]. Some studies have reported that mental health professionals’ attitudes toward people with mental illnesses are similar to—and occasionally more negative than—those of the general public; this is contrary to the common assumption that such professionals, as a result of their knowledge of mental illness and daily contact with people experiencing mental illness, have more favorable attitudes toward such people [8,9]. It has also been reported that university students majoring in health science often hold stigmatized views of people with mental illnesses, suggesting that the current curricula for healthcare providers do not positively influence students in this regard [10,11].

Several interventions have been employed to tackle stigma toward psychiatric illness, including education through lectures and case sessions, and the use of contact-based interventions that involved either direct or filmed contact [12,13]. Education is the most commonly implemented stigma-reduction approach and is mostly applied based on etiological models of psychiatric illness [14]. Conceptualizing mental disorders biologically can represent patients as being physiologically different from people without diagnosed mental disorders, and as being governed by genetic or neurochemical abnormalities instead of their own human agency; such perceptions can engender negative social attitudes and dehumanization [15,16]. Notably, biogenetic causal beliefs and diagnostic labeling are positively related to prejudiced views among the public, as well as fear and a desire for distance among the victims of such stigma [17]. The video approach is effective in improving attitudes toward people with mental illnesses and knowledge about mental illness [18,19], but its effects on actual behavior have not been fully confirmed [19]. The contents of the videos affect the effectiveness of stigma reduction [19,20]. Contact had a relatively stable effect on students’ attitudes and intended behavior that was stronger than the effect of short video interventions [21]. Contact and educational interventions have small-to-moderate immediate effects on stigma reduction [22]. To drastically reduce stigma, a new program is needed that emphasizes potential recovery from mental illness, as well as multiple types of social contact [23,24], collaborative working, the building of partnerships and networks, and the involvement with people with lived experiences of mental illness [25,26].

People with mental illnesses often internalize stereotypes about mental illness, a tendency known as self-stigma, which can cause them to experience a loss of self-esteem and self-efficacy [27]. Specifically, higher levels of internalized stigma are associated with lower levels of hope, empowerment, self-esteem, self-efficacy, quality of life, and social support [28]. Furthermore, among outpatients, loneliness, low social support, perceived stigma, experience of stigma, and anticipated stigma reportedly contribute to higher self-stigma [29]. Thus, reducing self-stigma is imperative; consequently, there is a need for strategies and interventions that empower people with mental illnesses to pursue their life goals [27]. Various interventions have been developed to help reduce self-stigma, including measures based on increasing the understanding of mental illness, providing psychoeducation, and applying cognitive behavioral therapy [30]. Corrigan et al. [27] recommended developing educational programs that teach basic social and coping skills required for personal competence in society. Such programs would allow people to demonstrate their capabilities in critical social situations, thereby increasing their self-esteem and self-perceived value in the community and subsequently reducing self-stigma.

“Co-production” means the establishment of a partnership between healthcare professionals and patients and the mobilization of patient resources to achieve ”value co-creation” between them [31]. Meanwhile, dialogue is a therapeutic process that allows all participants to interactively develop the understanding of an issue [32]. We believe that by combining co-production and dialogue in a new stigma-reduction program for both health science students and people with a mental illness, co-production of value will occur, which is not possible in conventional stigma-reduction programs, and that mutual understanding will be deepened. This should reduce participants’ stigma toward mental illness and the self-stigma of people with mental illnesses.

In this study, the Co-Production with Dialogue Program for Reducing Stigma (CPD-RS) was developed. This program targeted both university students majoring in health science and community-dwelling people with mental illnesses, aiming to reduce stigma toward people with mental illnesses among the former group and self-stigma among the latter group. The program aimed to encourage students and people with mental illnesses to recognize each other as dignified individuals who have similar life problems and use similar coping strategies. This study specifically aimed to test the effectiveness of the program in reducing stigma among both students and people with mental illnesses.

## 2. Methods

### 2.1. Design and Setting

This study featured a quasi-experimental pre- and post-test design and did not include a comparison group. It was conducted at Aino University, Japan, from October 2019 to January 2020.

### 2.2. Participants

The participants were university students majoring in health science and community-dwelling people with a mental illness. The students participating in this study were second-year students who attended a series of lectures on basic psychiatry and occupational therapy, but only had limited contact with people with mental illnesses. We did not recruit first-year students because their present level of education was regarded as similar to that of the general public. Third- and fourth-year students were excluded from this study because they had contact with patients with mental illnesses during their preclinical practice.

A class of 40 students is the general size in an occupational therapy department in Japanese universities. We recruited 40 students from a second-year occupational therapy class. Before enrolling in this study, the students received fifteen lectures (90 min per lecture) on the basic knowledge of psychiatry (psychiatric illness, symptoms, etiology, and psychiatric treatments) as basic subjects for occupational therapy. From baseline to one month after intervention, students did not have psychiatry lectures; instead, they had general medicine lectures on internal medicine, orthopedics, neurosurgery, etc. With an estimated participation rate of 80–90% among students, the number of students would result in three–four participants per group. In general, the optimal number of group members is three–eight for group work [33].

Thus, we aimed to recruit 20 people with mental illnesses living in the community, to ensure that two people with mental illnesses would be placed in each group. We created flyers for participation in the CPD-RS in collaboration with an institute of Support for Continuous Employment (Type B service) and a psychiatric clinic and asked the staff at each facility to post these flyers to solicit participation. The people with mental illnesses had diagnoses of either schizophrenia, schizotypal and delusional disorders (F2), or mood (affective) disorders (F3) per the International Classification of Diseases (ICD-10); at the time of the study, all of them were receiving Support for Continuous Employment (Type B services) or engaging in a psychiatric daycare treatment program associated with a psychiatric clinic. Interviews were conducted to confirm that the participants had no communication-related difficulties and understood the study’s purpose. The participants in this study were health science students and people with mental illnesses known to the examiner; therefore, the possible risk of examiner–subject bias cannot be denied.

### 2.3. Intervention

**Figure 1:**
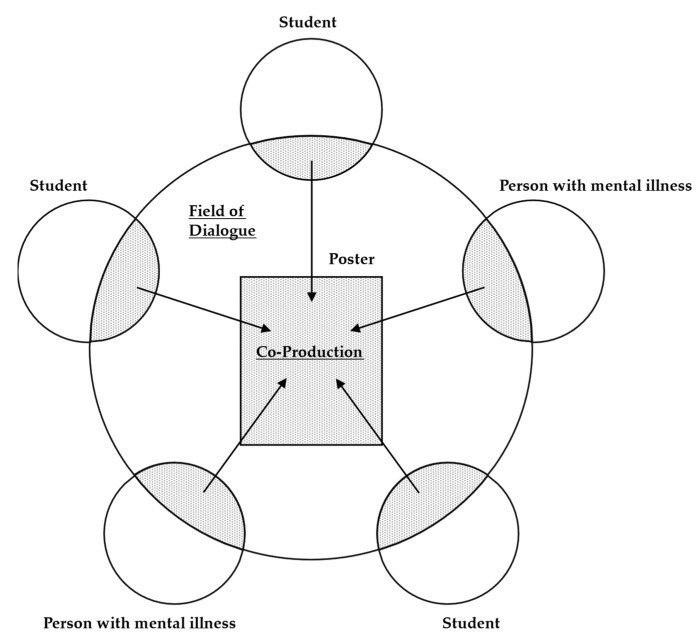
Co-Production with Dialogue Program for Reducing Stigma.

We designed and implemented the CPD-RS with the aim of promoting mutual understanding between the people with mental illnesses and the students through co-production with dialogue; we also aimed to reduce the students’ stigma toward people with mental illnesses and the self-stigma of the people with mental illnesses. The program was conducted face-to-face in a conference room at the university. We explained the program outline and schedule to both students and people with mental illnesses. In the CPD-RS, the students and the people with mental illnesses worked in groups to create posters concerning a specific theme. Figure 1 presents an overview of the CPD-RS. The themes for the co-production with dialogue were as follows: (1) coping with daily life and personal problems, (2) problems encountered in interpersonal relationships and means of dealing with them, and (3) how to have fun in daily life. The first author (E.N.) and coauthors (T.H. and M.T.), each of whom have over 10 years of experience in medical and/or rehabilitation practice and/or education, determined the themes using the principles of recovery-oriented mental health practice [34] and the recovery conceptual framework for personal recovery [35,36] as base material. The appropriateness of these themes was confirmed by two health science education professionals who were not involved in this study.

Note. Group members: university students (3 students) and people with a mental illness (2 people). Group members implemented co-production with dialogue and created posters. The themes for co-production with dialogue were as follows:

1. Coping with daily life and personal problems.
2. Problems encountered in interpersonal relationships and means of dealing with them.
3. How to have fun in daily life.

For each of the three themes, the students and the people with mental illnesses wrote their opinions on sticky notes and created posters while engaging in co-production with dialogue. The three principles of co-production with dialogue were: (1) listen to the other person until the end, (2) speak from your own experience, and (3) do not criticize others’ opinions. In the poster-production phase, which was based on co-production, participants considered the experiences and opinions they had written on the sticky notes and sought to identify similar opinions as well as the relationships between their opinions and those of the other members. In the co-production with dialogue process, the students and the people with mental illnesses shared their life experiences and tips on interpersonal relationships and life problems. Co-production with dialogue was conducted for 90 min. Two weeks later, each group presented their posters to the other groups. After the presentation, the group members reflected on the posters and their presentations. These group presentations were conducted over a 90 min period.

### 2.4. Data Collection

The participants’ demographic data (age, gender, diagnosis) were obtained through a questionnaire. The Devaluation Discrimination Scale (DDS), which is a 12-item self-administered questionnaire developed by Link [37], was used to assess the students’ stigma-related perceptions of mental illness and the self-stigma of the people with a mental illness. Participants were also administered a postintervention questionnaire that aimed to assess their experience of participating in the program. To ensure that participants’ responses were anonymous, identification numbers were used for all questionnaires, rather than names.

The DDS assesses the extent to which an individual believes that most people will devalue or discriminate against a patient with mental illness [37]. Respondents indicate the extent to which they agree or disagree with statements regarding most people devaluing current or former psychiatric patients by considering them failures, less intelligent than others, or individuals whose opinions need not be taken seriously [2]. Moreover, the scale includes items that assess perceived discrimination by most people in jobs, friendships, and romantic relationships [2]. Respondents are initially presented with a description of the scale; thereafter, they report their level of agreement with the 12 statements concerning devaluation and discrimination against people with mental illnesses [2]. Responses are provided on a four-point scale (Japanese version; 1 = “strongly disagree,” 2 = “slightly disagree,” 3 = “slightly agree,” and 4 = “strongly agree”) [38]. Some statements in the questionnaire are reverse-scored (R). The scale can be administered to both the general public and people with mental illnesses [2]. Although self-stigma has been measured in various ways in the literature (e.g., Link et al. and Livingstone et al. [2,28], in this study, we have utilized the DDS in accordance with the study by Link et al.) [37]. The Japanese version of the DDS is, reportedly, reliable and valid (Cronbach’s α = 0.85, θ = 0.85) [38]. The DDS scores of people with mental illnesses in the present study were similar to the mean scores of people with mental illnesses in a Japanese study by Shimotsu et al. [38]. The DDS was administered to the participants on the following four occasions: one month before the intervention (“baseline”; October 2019), immediately before the intervention (“pre-intervention”; November 2019), immediately after the intervention (“post-intervention”; December 2019), and one month after the intervention (“one month after intervention”; January 2020).

To assess participants’ program-participation experience, a postintervention questionnaire was administered. This questionnaire contained six items (focusing on enjoyment of the program, the level of self-expression and mutual interaction in the program, similarities among participants’ life experiences and tips, influences of one’s own experiences, influences of others’ experiences, and willingness to tell others about one’s experience of the program) that were answered using a five-point Likert scale (1 = “very negative,” 5 = “very positive”), and also contained an open-ended question (“How did you feel about participating in the program?”).

### 2.5. Statistical Analysis

Statistical tests were performed by comparing the total DDS scores at four time points (October 2019, November 2019, December 2019, and January 2020). The Shapiro–Wilk test was used to examine whether the acquired data were normally distributed. The comparisons of the four sets of DDS scores, from October to January, were conducted by applying a repeated-measures analysis of variance (ANOVA). Tukey’s multiple comparison test was performed when cases of significant differences emerged through this comparison. Nonparametric tests were performed if the data were not normally distributed. Bonferroni corrections were performed for the results of multiple comparisons and for comparisons of DDS sub-items. For the postintervention questionnaire, the medians and interquartile ranges were calculated for each item (except for the open-ended question). IBM SPSS Statistics for Windows, version 26 (IBM Corp., Armonk, NY, USA) was used to perform statistical analyses. The free text provided in the responses to the open-ended question was summarized.

### 2.6. Ethics and Consent

This study was conducted in accordance with the stipulations of the Declaration of Helsinki. Approval was obtained from the Research Ethics Committee of Aino University (approval No. 2018-21). Individuals who expressed a wish to participate were informed that participation was voluntary and that they could cancel their participation at any time. The research commenced after written consent was obtained from all participants.

## 3. Results

### 3.1. Participants

A total of 48 people participated in the CPD-RS—28 students and 20 community-dwelling people with a mental illness. Based on the ICD-10 classification, among the participants with a mental illness, 9 had a diagnosis of F2 and 11 had a diagnosis of F3. Participants’ demographic characteristics are shown in Table 1. All participants were randomly assigned to 10 groups. Each group comprised three students and two people with a mental illness, except for one group, which comprised four students and two people with a mental illness. None of the students or people with mental illnesses dropped out of the program.

### 3.2. Multiple Comparisons from Baseline to One Month after Intervention

The DDS scores at baseline, preintervention, postintervention, and one month after the CPD-RS intervention were compared; all 28 participating students and all 20 people with a mental illness responded to the DDS at each of the four time points. The Shapiro–Wilk test showed that, for all DDS scores, both those of the students and those of the people with mental illnesses, the data were normally distributed; thus, parametric statistics were applied (Table 2).

In students, a repeated-measures ANOVA of DDS scores at the four time points revealed a significant difference (*p* = 0.00014, effect size, η^2^ = 0.06; Table 2). Tukey’s multiple comparison test was performed for the four time points in students, and Bonferroni’s correction was applied to the results of these multiple comparisons (Table 3). As a result, the acceptable level of significance was set at 0.0125. No significant difference was found in students’ DDS scores between baseline and preintervention (Table 3). We observed a tendency to decrease between baseline and postintervention (mean difference −2.61, *p* = 0.025), and between baseline and one month after intervention (mean difference −2.07, *p* = 0.108). In DDS scores between pre- and postintervention, there was a significant difference (*p* = 0.0004; effect size, Δ = −0.55), and likewise between preintervention and one month after intervention (*p* = 0.003; effect size, Δ = −0.54). There was no significant difference in total DDS students’ scores between postintervention and one month after intervention (Table 3). The DDS score of students at baseline was similar to that in a previous report [38]. For the DDS subitems, comparisons were conducted between the pre- and postintervention scores and between the preintervention and one month postintervention scores; this was performed using Wilcoxon’s signed-rank test. Bonferroni’s correction was applied to the results. Consequently, the acceptable level of significance was set at 0.00416. The results for the comparison between the pre- and postintervention scores showed that scores for two items reduced significantly after the intervention, namely Question 5: “Most people feel that entering a mental hospital is a sign of personal failure” (*p* = 0.003; effect size, r = 0.56) and Question 6: “Most people would not hire a former mental patient to take care of their children, even if he or she has been well for some time” (*p* = 0.0013, r = 0.61; Table 4). There were no significant changes among the other subitems.

In contrast, we could not observe any significant differences in the DDS total scores of people with mental illnesses across the four time points (Table 2). The DDS scores of people with mental illnesses at baseline were similar to those in a previous report [38], suggesting that the people with mental illnesses who participated in this study did not show bias and that there was no ceiling or floor effect.

### 3.3. Participants’ Evaluation of the Intervention Program

Overall, 16 students and 20 people with a mental illness responded to the postintervention questionnaire. Mutual cooperation was indirectly measured by using the questionnaire. The total scores (presented as median [interquartile range]) for all six items were 4 (4–5) for the students and 3.5 (3–4.5) for the people with a mental illness (Table 5).

In the free-text responses to the open-ended question, both students and people with mental illnesses stated that they enjoyed participating in the program together. Representative comments from the students are: “I was able to talk about life and life problems with people with mental illnesses,” “I gained the understanding that students and the people with mental illnesses experience similar problems,” “I was surprised that we had many things in common, and that our problems were not so different,” and “The people with mental illnesses had constructive opinions.” Meanwhile, representative comments from the people with mental illnesses are: “I felt that both young and not-so-young people experience the same problems with relationships,” “It was good to be able to talk with students who are around my own age,” and “It was good to be able to talk with students who are as old as my own children.”

## 4. Discussion

This study aimed to develop the CPD-RS to reduce stigma and examine its effectiveness for both health science students and people with a mental illness. After engaging in the CPD-RS program, the participating students’ total DDS scores showed a significant decrease when compared to their preintervention scores, and this reduction effect was sustained for at least one month. These results suggest that the CPD-RS is effective for reducing students’ stigma toward people with mental illnesses.

During the two months prior to the intervention, the students received lectures on psychiatric disorders and biogenetic epidemiology. However, these lecturers did not induce a significant reduction in the students’ total DDS scores. This is similar to previous studies indicating that providing biogenetic explanations for mental disorders is unlikely to reduce stigma [39]. Moreover, holding biogenetic causal beliefs concerning mental disorders is associated with a greater likelihood of stigma-based perceptions and/or negative attitudes toward mental illness [40]. Combined with previous reports, our results suggest that providing knowledge of psychiatric disorders and biogenetic explanations of these disorders is insufficient for stigma reduction.

The CPD-RS intervention caused a decrease in students’ total DDS scores. This program comprised co-production with dialogue. The cause of the students’ reduced DDS scores may be associated with the program’s characteristics. On investigating the students’ DDS subitem scores, we found significant postintervention reductions in scores for two items: “Most people feel that entering a mental hospital is a sign of personal failure” and “Most people would not hire a former mental patient to take care of their children, even if he or she has been well for some time.” These results suggest that the interventions can foster students’ respect for the dignity of people with mental illnesses and belief in their recovery. The CPD-RS comprises more than just contact with people with mental illnesses. It is an intervention program that establishes partnerships through co-production [31], and it allows all participants to interactively learn about skills and tips for solving problems in their lives through dialogue [25]. The students’ experiences in this program may have caused them to feel closer to people with mental illnesses. Knaak et al. [25] mentioned that stigma-reduction programs should emphasize the possibility of recovery from mental illness and that such programs should be “person-centered” and feature multiple forms of social contact. The CPD-RS met these requirements, resulting in its observed effectiveness.

Mehta et al. [41] reported—after conducting a systematic review of interventions for reducing mental health-related stigma and discrimination—that the maintenance effect of attitudinal and behavioral changes toward people with mental disorders is marginal. However, in the present study, the students’ total DDS scores were not only reduced at the postintervention time point but also at one month after the intervention. This suggests that the stigma-reduction effect of the CPD-RS persists for at least one month.

For the people with a mental illness who participated in this study, there was no significant change in total DDS scores after the intervention, suggesting that the CPD-RS does not improve self-stigma. Various studies have presented recommendations regarding requisites for improving self-stigma; for example, Dubreucq et al. [29] suggested that such programs should be recovery-oriented, while Díaz-Mandado and Perianez [42] suggested that mutual cooperation between participants is needed to foster the understanding of each person’s strengths, needs, and goals. The CPD-RS is recovery-oriented and promotes mutual cooperation; however, to reduce self-stigma in people with mental illnesses, extending the duration and increasing the frequency of this intervention may be necessary. Moreover, ensuring the program structure such that the participants can provide positive feedback to others is important.

In the questionnaire concerning their experiences of participating in the CPD-RS, which was administered two months after the intervention, the students and the people with mental illnesses reported that they enjoyed the CPD-RS and felt that it facilitated self-expression and interaction. They also stated that they were able to find commonalties between themselves and the other members with respect to approaches for solving life problems. Moreover, they stated that they would like to share their experiences of participating in this program with others. These findings suggest that the intervention helped remove the division between “us” and “them”—an outcome noted as important by Knaak et al. [25]. Finally, none of the students or the people with mental illnesses who participated in the CPD-RS withdrew from the program due to perceived burden, invasiveness, or stress; this, along with the above results, suggests that the intervention program is feasible, useful, and safe.

There are several limitations to this study. First, since the research method was quasi-experimental, with a pre- and post-test design, a control group could not be established. Second, the number of participants was small. Third, the participants were health science students and people with mental illnesses known to the examiner, so there is a possible risk of examiner–subject bias. Fourth, considering that the response rate for the postintervention questionnaire was only 57% for students, because the collection coincided with the end of the semester and the following vacation period, we cannot rule out the possibility that the questionnaire results were influenced by subject selection bias. Fifth, as the stigma-reducing effect of the intervention was only followed up to one month after the intervention, further research is needed to evaluate the maintenance effect beyond one month. Therefore, a longitudinal study design featuring a higher number of participants and a control group will be needed in the future; in this study, it will also be necessary to prevent the risk of examiner–subject biases by incorporating blinding in the study design. Last, the general public also tends to exhibit a strong stigma against people with mental illnesses; thus, in future research, expanding the target population will be necessary to determine whether the CPD-RS is effective in a general context. The concept of internalized stigma has attracted attention toward the self-stigma of people with mental illnesses [28]. In addition to the perceived stigma, as assessed by the DDS, further studies must focus on internalized stigma.

## 5. Conclusions

The CPD-RS—an intervention method based on co-production with dialogue—reduces students’ stigma toward people with a mental illness, with the effect lasting at least one month after the intervention and fostering mutual understanding between these groups. The results also suggest that the CPD-RS can be implemented safely.

## Figures and Tables

**Table 1:** Characteristics of the CPD-RS participants.

Variables	Students (*n* = 28)	People with a Mental Illness (*n* = 20)
Sex		
Male (%)	11 (39%)	13 (65%)
Female (%)	17 (61%)	7 (35%)
Age, years ^a^	20.23 (1.26)	52.25 (9.78)
Diagnosis		
F2	NA	9
F3	NA	11

^a^ Age is represented as: mean (standard deviation).

**Table 2:** Total DDS scores of students and people with mental illnesses across four time points.

		Total DDS Scores ^a^			*p*-Value ^b,^*
	Baseline	Preintervention	Postintervention	One Month after Intervention	
Students (*n* = 28)	29.04 (6.29)	30.21 (6.57)	26.43 (6.29)	26.96 (5.37)	0.00014 *
People with a mental illness (*n* = 20)	31.70 (5.52)	31.40 (6.12)	30.05 (7.20)	31.0 (7.03)	0.61

DDS: Devaluation Discrimination Scale. ^a^ Total DDS scores are represented as mean (standard deviation). ^b^ *p*-values were calculated through a repeated-measures analysis of variance (* *p*  <  0.05).

**Table 3:** Multiple comparisons of the students’ total DDS scores across the four time points (*n* = 28).

Period	Comparison	Mean Difference	95% Confidence Intervals for Differences		*p* Value ^a^
			Lower	Upper	
Baseline	Preintervention	1.18	−3.55	1.19	0.563
	Postintervention	−2.61	0.24	4.98	0.025
	One month after intervention	−2.07	−0.30	4.44	0.108
Preintervention	Postintervention	−3.79	1.42	6.16	0.0004 *
	One month after intervention	−3.25	0.88	5.62	0.003 *
Postintervention	One month after intervention	0.54	−2.91	1.83	0.934

^a^*p*-values were calculated using Tukey’s multiple comparison test (*p*  <  0.05). * The Bonferroni method was used, and the *p*-values were adjusted to 0.0125.

**Table 4:** Comparison of students’ DDS subitem scores between pre- and postintervention periods (*n* = 28).

DDS Item	Preintervention ^a^	Postintervention	*p*-Value ^b^
5. Most people feel that entering a mental hospital is a sign of personal failure.	2 (2–3)	2 (1–2.8)	0.003 *
6. Most people would not hire a former mental patient to take care of their children, even if he or she has been well for some time.	3 (2–3)	2 (1.3–3)	0.0013 *

^a^ DDS subitem values are presented as median (interquartile range). ^b^ Comparisons were conducted using Wilcoxon’s signed-rank test. * The Bonferroni method was used, and the *p*-values were adjusted to 0.00416. These two subitems were scored in the questionnaire as follows: 1 = “strongly disagree,” 2 = “slightly disagree,” 3 = “slightly agree,” 4 = “strongly agree”.

**Table 5:** Participants’ evaluations of the CPD-RS.

Questionnaire	Students (*n* = 16)	People with a Mental Illness (*n* = 20)
1. Did you enjoy participating in this activity?	4 (4–5)	4(3–5)
2. Did you have sufficient time to talk about your own experiences?	4 (3.8–5)	3.5 (3–4.3)
3. Did you find that your responses and methods of coping with life problems are similar to those of many other group members?	4 (4–5)	3.5 (3–4.3)
4. Did you feel that your experiences were helpful to other people?	4 (3–4)	3 (3–4)
5. Did you feel that the experiences of others were helpful to you?	4.5 (4–5)	4 (3–4.5)
6. Would you like to share the experiences you had in this class with someone else?	4 (4–5)	3 (3–4.8)
All items	4 (4–5)	3.5 (3–4.5)

Values are represented as: median (interquartile range). The six items were scored using a five-point Likert scale (1 = “very negative,” 2 = “negative,” 3 = “neither,” 4 = “positive,” 5 = “very positive”).

## Data Availability

The datasets used and/or analyzed during the current study are available from the corresponding author on reasonable request.
